# Acute Hemoptysis Caused by Eroding Spinal Fusion Hardware Into the Left Lower Lobe of the Lung

**DOI:** 10.7759/cureus.49918

**Published:** 2023-12-04

**Authors:** Christian S Bury, Salomey O Antwi, Ilya Fonarov, Damian Casadesus

**Affiliations:** 1 Internal Medicine, Jackson Memorial Hospital, Miami, USA; 2 Internal Medicine, St. George's University School of Medicine, Great River, USA; 3 Primary Care, Orlando College of Osteopathic Medicine, Orlando, USA

**Keywords:** adolescent idiopathic scoliosis (ais), bronchoscopy, bronchial embolization, spinal rod, acute hemoptysis

## Abstract

Hemoptysis can occur in rare cases as a late complication of anterior approach spinal rod surgery in the spine. Our patient presented with hemoptysis. At age 14, he underwent an anterior approach spine surgery for scoliosis. He underwent bronchoscopy, and a round serrated metal object was visible in the left lower lobe. Multiple attempts were unsuccessful in retrieving the metal object. A review of the imaging did not show obvious penetration of the spinal instrumentation into the lung; however, the metallic object was believed to be a round serrated metal object from the fusion spinal hardware. Due to low lung function and the risk of injuring the surrounding tissue, the patient was not deemed a candidate for lobectomy or removal of the hardware. Instead, the patient underwent a left bronchial arterial embolization (BAE) procedure, which successfully stopped the hemoptysis.

## Introduction

Non-life-threatening hemoptysis typically does not cause significant hemodynamic instability or respiratory failure. Common causes of non-life-threatening hemoptysis include foreign body aspiration, acute bronchitis, bronchiectasis, and malignancy. Management of the underlying etiology is important to prevent the recurrence of hemoptysis. In acute situations, nonsurgical control of the bleeding is preferred. Diagnostic and therapeutic bronchoscopy is employed first, but its overuse has been noted particularly in younger patients with low visualization of the site of bleeding, such as in this case [1,2]. Bronchial arterial embolization (BAE) is used to treat life-threatening hemoptysis as well as non-life-threatening hemoptysis in patients who cannot tolerate surgery. BAE is particularly useful when an involved artery is noted on computed tomography angiography (CTA). Surgery is reserved for patients whose medical treatment and embolization are not effective [1-3].

## Case presentation

A male in his 40s with a past medical history of surgical treatment for adolescent idiopathic scoliosis using an anterior approach to the chest at age 14, gastroesophageal reflux disease, Barrett's esophagus, Wolff-Parkinson-White syndrome, and dyslipidemia presented to the emergency room with acute onset of hemoptysis. He reported having the urge to cough before frank hemoptysis occurred. The patient reported a previous similar urge with minimal hemoptysis a few months prior to this presentation. He smoked one pack of cigarettes per day for 20 years, but he stopped smoking eight years prior to this presentation.

On physical examination, the vital signs were blood pressure of 136/66 mmHg, temperature of 36.6°C, heart rate of 78 beats per minute, respiratory rate of 18 breaths per minute, and oxygen saturation of 99% on ambient air. Respirations were unlabored, and the lungs were clear to auscultation bilaterally. Cardiovascular, neurological, and abdominal examinations were unremarkable.

Investigations

**Figure 1:**
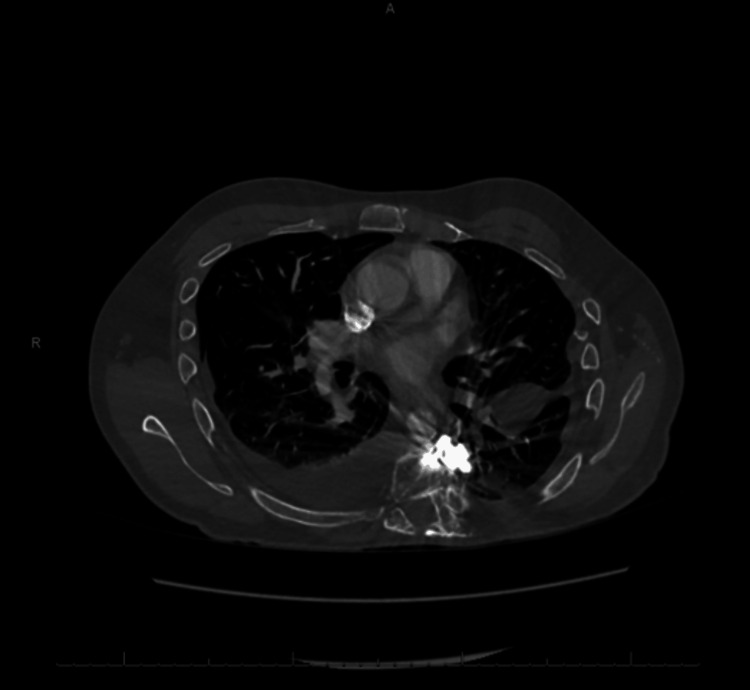
CTA of the chest revealing the fusion orthopedic hardware component in the anterior prevertebral space abutting the pleura (axial view) CTA: computed tomography angiogram

**Figure 2:**
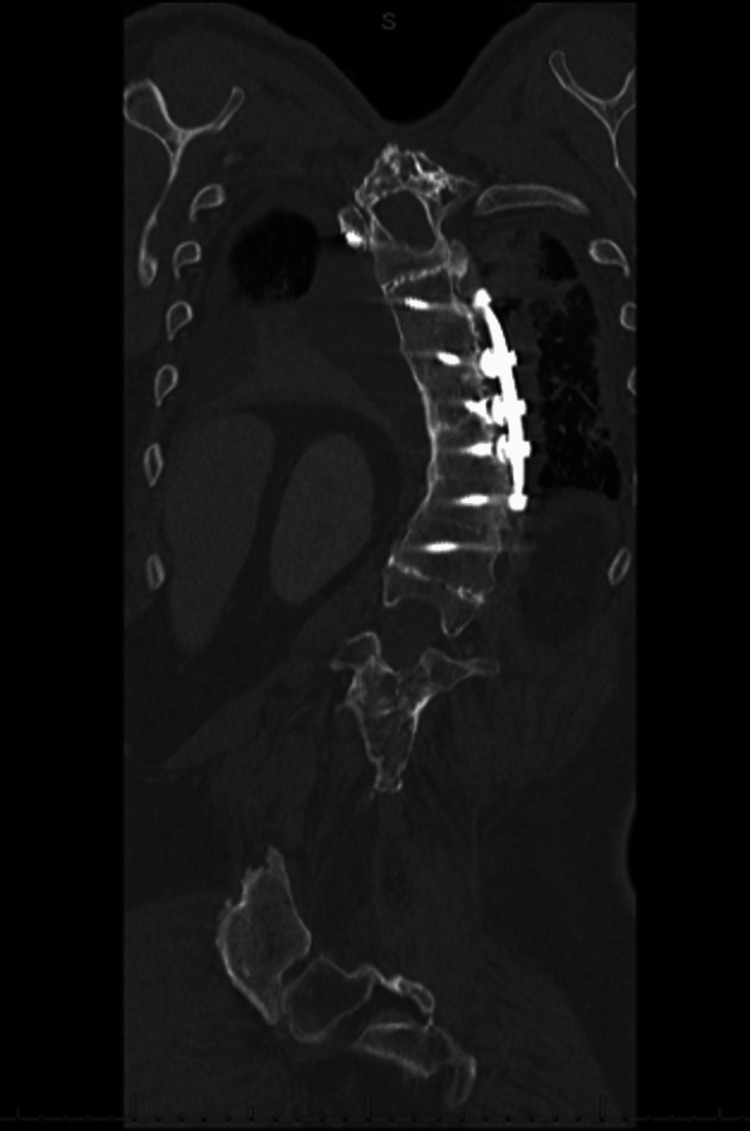
CTA of the chest revealing intact fusion hardware close to the left lung (coronal view) CTA: computed tomography angiogram

Relevant laboratory studies for the patient were all within normal limits and are shown in Table 1. Pulmonary mycobacterial and fungal infection testing was negative as was SARS-CoV-2 RNA RT-PCR. Sputum for bacterial culture was positive for methicillin-sensitive *Staphylococcus aureus* (MSSA). Chest X-ray on admission showed increased perihilar and interstitial markings. Computed tomography angiography (CTA) of the chest revealed no pulmonary embolism and diffuse bilateral ground glass opacities with superimposed tree-in-bud opacities throughout the lungs. In addition, the fusion orthopedic hardware component in the anterior prevertebral space was abutting the pleura but did not appear to penetrate the lung (Figures 1, 2). Pulmonary function testing (PFT) revealed a severe restrictive ventilatory defect with moderate diffusion capacity impairment.

**Table 1:** Relevant laboratory results upon investigation WBC: white blood cell count, Hb: hemoglobin, Hct: hematocrit, Plt: platelet count, PT: prothrombin time, APTT: activated partial thromboplastin time

Test	Patient's value	Normal range
WBC	7.3 × 10^3^/µL	4.5-11 × 10^3^/µL
Hb	14.5 g/dL	12-16 g/dL
Hct	45.80%	36%-46%
Plt	247 × 10^3^/µL	150-450 × 10^3^/µL
PT	14.5 seconds	10-13 seconds
APTT	34 seconds	25-35 seconds

**Figure 3:**
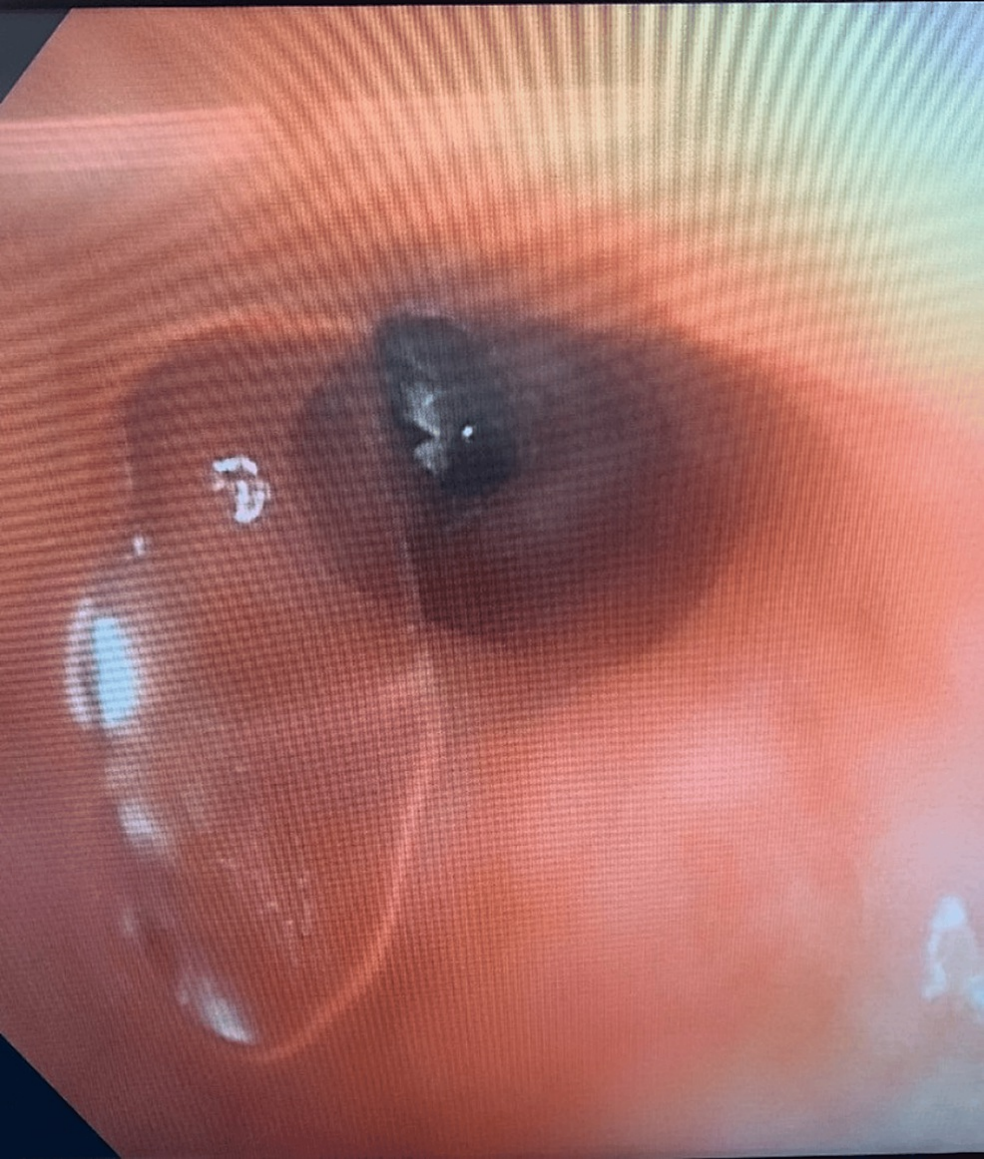
Bronchoscopy image revealing metallic round serrated object in the anteromedial basal segment of the left lower lobe

He underwent bronchoscopy, and a round serrated object was visible distal in the anteromedial basal segment of the left lower lobe behind a granuloma (Figure 3). An attempt to retrieve it with a snare and forceps was unsuccessful. The patient was treated with intravenous piperacillin-tazobactam 3.375 g for a total of 10 days for MSSA pneumonia. The patient was transferred to a tertiary care hospital for a thoracic surgery evaluation. The metallic object was suspected to be related to the orthopedic apparatus, and a repeat bronchoscopy was attempted to retrieve the metallic object. The object was unable to be retrieved and was believed to be an eroding spinal screw from his spinal instrumentation.

Interventions for the hemoptysis were considered, either removing the orthopedic hardware or resecting the lung. From a pulmonary perspective, the patient was at high risk for complications including fracture, loosening of the screw, and infections postoperatively if he underwent lung resection, and therefore, surgery was determined not to be viable [4]. After further discussion with cardiothoracic and orthopedic surgery, the next best potential intervention was determined to be an attempt to embolize the posterior segment of the left lower lobe by interventional radiology. Left BAE using 500-700 micron embospheres was performed.

Outcome and follow-up

Post-embolization, the patient denied any further hemoptysis even after ambulation, which initially triggered the hemoptysis. No further surgical interventions were necessary, and the patient was discharged. The patient was treated with intravenous piperacillin-tazobactam 3.375 g for a total of 10 days for MSSA pneumonia and improved clinically. Follow-up with pulmonology and primary care was provided.

## Discussion

Acute non-life-threatening hemoptysis is frequently caused by necrotizing pneumonia, active tuberculosis, mycetomas, bronchiectasis, and malignancy [1-3,5]. Our patient was initially treated with intravenous piperacillin-tazobactam for MSSA pneumonia based on bronchial culture and imaging results. Tuberculosis and *Aspergillus* were considered in the differential diagnosis, but all studies were negative. The patient denied any ingestion or aspiration of a foreign body. Unfortunately, our patient developed acute hemoptysis secondary to perforation in his left lower bronchus by the spinal hardware.

Our patient had been without any significant complications for over 30 years following anterior spinal instrumentation surgery to correct his scoliosis. It is known that the anterior approach has an unfavorable effect on lung function, and anterior spinal surgery necessitating a thoracotomy or thoracoscopy resulted in a fall in lung volumes [6-8]. Surgical removal of the orthopedic hardware was considered, but it was felt to be too high risk. The procedure would require an open thoracotomy approach and the use of a metal cutting burr in a very sensitive area. The vital structures nearby could be damaged by metal debris and the cutting bit. It was felt that removing the hardware would be more morbid and pose a higher risk than a partial lobectomy. Partial lobectomy was considered, but the patient had a severe restrictive ventilatory defect on PFT. He was deemed a poor surgical candidate. There is a reported case of hemoptysis in a patient with scoliosis who had undergone anterior lateral spinal fusion [9]. In that case, the imaging of the chest revealed penetration of the spinal rod into the right upper lobe of the lung. Due to the high risk of complications, the patient underwent extensive surgery to remove the spinal rod.

In contrast, our patient underwent BAE. It is a relatively safe and effective method for immediate control of hemoptysis from benign and malignant causes, and complications are rare. The long-term success rate of BAE is dependent on the underlying cause of the hemoptysis. A low clinical success rate was noted in patients with unstable hemodynamics and coagulopathy, while multiple vessel embolization was associated with higher clinical success. Because the bleeding recurrence rate is high in patients with lung cancer or idiopathic bronchiectasis, surgery should be considered in these patients following initial stabilization by bronchial artery embolization [10-12]. Serious adverse complications of BAE include transverse myelitis, bronchial infarction, esophagobronchial fistula, ischemic colitis, transient cortical blindness, and stroke. The most feared complication of BAE is spinal cord ischemia due to the inadvertent embolization of a spinal artery, fortunately occurring in <5% of cases in most studies. Minor complications include transient chest pain and dysphagia, with transient chest pain being the most common [13]. Although the long-term outcome in some patients is not optimal, BAE may be the only lifesaving treatment option in patients who are poor surgical candidates.

## Conclusions

This case exemplifies the unfavorable effect on lung function and potential complications that anterior approach spinal surgery for scoliosis can have on a patient decades after the procedure is completed. Our patient experienced non-life-threatening hemoptysis due to an eroding spinal rod screw. Because of the precarious location of the bleed and the patient's severe restrictive ventilatory defect with moderate diffusion capacity impairment, he was deemed unfit for an open thoracotomy to remove the orthopedic hardware or a partial lobectomy, respectively. BAE was the treatment of choice in this case due to the risks of thoracic surgery in a patient with compromised pulmonary function.
